# An Efficient Feature Subset Selection Algorithm for Classification of Multidimensional Dataset

**DOI:** 10.1155/2015/821798

**Published:** 2015-09-28

**Authors:** Senthilkumar Devaraj, S. Paulraj

**Affiliations:** ^1^Department of Computer Science and Engineering, University College of Engineering, Anna University, Tiruchirappalli, Tamil Nadu, India; ^2^Department of Mathematics, College of Engineering, Anna University, Tamil Nadu, India

## Abstract

Multidimensional medical data classification has recently received increased attention by researchers working on machine learning and data mining. In multidimensional dataset (MDD) each instance is associated with multiple class values. Due to its complex nature, feature selection and classifier built from the MDD are typically more expensive or time-consuming. Therefore, we need a robust feature selection technique for selecting the optimum single subset of the features of the MDD for further analysis or to design a classifier. In this paper, an efficient feature selection algorithm is proposed for the classification of MDD. The proposed multidimensional feature subset selection (MFSS) algorithm yields a unique feature subset for further analysis or to build a classifier and there is a computational advantage on MDD compared with the existing feature selection algorithms. The proposed work is applied to benchmark multidimensional datasets. The number of features was reduced to 3% minimum and 30% maximum by using the proposed MFSS. In conclusion, the study results show that MFSS is an efficient feature selection algorithm without affecting the classification accuracy even for the reduced number of features. Also the proposed MFSS algorithm is suitable for both problem transformation and algorithm adaptation and it has great potentials in those applications generating multidimensional datasets.

## 1. Introduction

The multidimensional classification problem has been a popular task, where each data instance is associated with multiple class variables [1]. High-dimensional datasets contain irrelevant and redundant features [2]. Feature selection is an important preprocessing step in mining high-dimensional data [3]. Time complexity is high for selecting the subset of features and for further analysis or to design the classifier if the number of features and targets (class variables) in the dataset is large. Computational complexity is based on three factors: number of training examples “*m*,” dimensionality “*d*,” and number of possible class labels “*q*” [4, 5].

The prime challenge for a classification algorithm is that the number of features is very large, whilst the number of instances is very small. A common approach to this problem is to apply a feature selection method in a preprocessing phase, that is, before applying a classification algorithm to the data, in order to select a small subset of relevant features for microarray data classification (high-dimensional data) [6, 7]. Multidimensional data degrade the performance of the classifiers and reduce the classifier accuracy and processing this data is too complex by traditional methods and needs a systematic approach [8]. Therefore, mining the multidimensional dataset is a challenging task among the recent data mining researchers.

Most of the proposed feature selection algorithms support only single-labelled data classification [9, 10]. The related feature selection algorithms do not fit into those applications generating multidimensional datasets [9]. The effective feature selection algorithm is an important task for efficient machine learning [11]. Feature selection in the multidimensional is the challenge task. The solution space, which is exponential in the number of target attributes, becomes enormous, even with a limited number of target attributes. The relationships between the target attributes can add a level of complexity that needs to be taken into account [12].

*χ* ^2^ statistic is used to rank the features of high-dimensional textual data by transforming the multilabel dataset into the single label classification using label powerset transformation [13]. The chi-square test is not suitable in determining the good correlation between the decision classes and features. Also, it is not suitable for the high-dimensional dataset [14]. Pruned problem transformation is applied to transform multilabel problem to single label and greedy feature selection employed by considering the mutual information [15]. REAL algorithm is employed for selecting the significant symptoms (features) for each syndrome (classes) in the multilabel dataset [16]. Classifier built from the MLD is typically more expensive or time-consuming with multiple feature subsets. It discusses the future works related to multidimensional classification such as studying different single-labelled classifiers and feature selection [1]. A genetic algorithm is used to identify the most important feature subset for prediction. Principal component analysis is used to remove irrelevant and redundant features [17].

In multidimensional learning tasks, where there are multiple target variables, it is not clear how feature selection should be performed. Limited research is only available on multilabel feature selection [9]. Therefore, we are in need of a robust feature selection technique for selecting the significant single subset of features from the multidimensional dataset. In this paper, an efficient feature selection algorithm is proposed for the multidimensional dataset (MDD).

The rest of this paper is organized as follows.  Section 2 briefly presents the basics of multidimensional classification and addresses the importance of data preprocessing.  Section 3 describes the proposed multidimensional feature subset selection (MFSS) which is based on weight of feature-class interactions.  Section 4 presents the experimental results and analysis to evaluate the effectiveness of the proposed model.  Section 5 concludes our work.

## 2. Preliminaries

This section presents some basic concepts of multidimensional classification and the importance of preprocessing in data mining.

### 2.1. Multidimensional Paradigm

**Figure 1:**
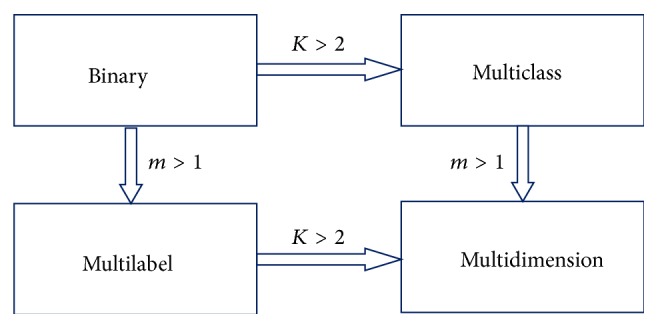
The relationship among different classification paradigms.

In general, the multidimensional dataset contains “*n*” independent variables and “*m*” dependent variables. Each instance is associated with multiple class values. The classifier is built from a number of training samples.  Figure 1 shows the relationship between different classification paradigms, where “*m*” is the number of class variables and “*K*” is the number of values for each of the “*m*” variables. Multidimensional classification assigns each data instance to multiple classes. In multidimensional classification, the problem is decomposed into multiple, independent classification problems, aggregating the classification results from all the independent classifiers; that is, one single-dimensional multiclass classifier is applied to each class variable, called problem transformation [1].

### 2.2. Multidimensional Classification

Multilabel classification (MLC) refers to the problem of instance labelling where each instance may have more than one correct label. Multilabel classification has recently received increased attention by researchers working on machine learning and data mining. Multilabel classification is becoming increasingly common in modern applications For example, a news article could belong to multiple topics, such as politics, finance, and economics, and also could be related to China and the USA as the regional categories. Typical examples include medical diagnosis, gene/protein function prediction and document (or text) categorization, multimedia information retrieval to tag recommendation, query categorization, gene function prediction, medical diagnosis, drug discovery, and marketing [18–21].

Traditional single-label classification algorithms refer to classification tasks that predict only one label. The basic algorithms are generally known as single-label classification and it is not suitable for the data structures found in real world applications. For example, in medical diagnosis, a patient may be suffering from diabetes and prostate cancer at the same time [18, 22].

Research on MLC has received much less attention compared to single-labelled classification. MLC problem is decomposed into multiple, independent binary classification problems and determines the final labels for each data point by aggregating the classification results from all the binary classifiers [23]. Due to its complex nature, the labelling process of a multilabel data set is typically more expensive or time-consuming compared to single-label cases. Learning effective multilabel classifiers from a small number of training instances is important to be investigated [8].

### 2.3. Handling Missing Values

Raw data collected from different sources in different format are highly susceptible to noise, irrelevant attributes, missing values, and inconsistent data. Therefore, data preprocessing is an important phase that helps to prepare high quality data for efficient data mining in the large datasets. Preprocess improves the data mining results and ease of the mining process. Missing values exist in many situations, where there are no values available for some variables. Missing values affect the data mining results. Therefore, it is important to handle missing values to improve the classifier accuracy in data mining tasks [24–27].

### 2.4. Feature Selection

Feature selection [FS] is an important and critical phase in pattern recognition and machine learning. This task aims to select the essential features to discard the less significant features from the analysis. It is used to achieve various objectives: reducing the cost of data storage, by facilitating data visualization, reducing the dimension of the dataset for the classification process in order to optimize the time, and improving the classifier accuracy by removing the redundant and irrelevant variables [28–30].

It is classified into three main categories: filters, wrappers, and embedded methods. In the filter method, selection criterion is independent of the learning algorithm. On the other hand, the selection criterion of the wrapper method depends on the learning algorithm and uses its performance index as the evaluation criterion. The embedded method incorporates feature selection as part of the training process [28–30].

## 3. Proposed Multidimensional Feature Subset Selection Algorithm

**Figure 2:**
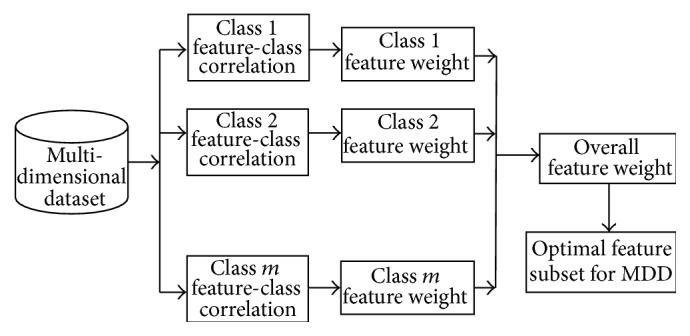
Proposed MFSS for multidimensional dataset.

In this section, the proposed algorithm for selecting the single subset of features from the MDD is presented. The block diagram of the proposed MFSS is shown in Figure 2.

MFSS has three phases. In the first phase, calculate the feature-class correlation, and assign weight for the features based on the feature-class correlation for each class. In the second phase, aggregate the results of feature weight of each class using proposed overall weight. In the third phase, select the optimal feature subset based on the proposed overall weight for further analysis or to build classifier. The proposed algorithm is developed from the correlation based attribute evaluation. A proposed MFSS algorithm for MDD is shown as follows.

**Algorithm 1 (multidimensional feature subset selection (MFSS)).**

 

*Input*. There is multidimensional dataset (MDD).

*Output*. Optimal single unique subset of “*s*” number of features from “*l*” features: *s* < *l*.

*Step 1*. Compute Pearson's correlation between feature and class using the equation

(1) $$\begin{array}{c} r_{f_{j}c_{i}}=\frac{n\left(\sum f_{jk}c_{ik}\right)-\left(\sum f_{jk}\right)\left(\sum c_{ik}\right)}{\sqrt{\left[n\sum {f_{jk}}^{2}-{\left(\sum f_{jk}\right)}^{2}\right]\left[n\sum {c_{ik}}^{2}-{\left(\sum c_{ik}\right)}^{2}\right]}}, \end{array}$$

where

 
*c*_*i*_—*i*th class *i* = 1 ⋯ *m* “*m*” is the number of classes,
 
*f*_*j*_—*j*th feature*j* = 1 ⋯ *l* “*l*” is the number of features,
 “*n*” is the number of observations, and
 
*r*_*f*_*j*_*c*_*i*__ is the (Pearson's correlation) between the *j*th feature and *i*th class.

Pearson's Correlation between the *j*th feature and *i*th class is represented as (*l* × *m*) matrix having “*l*” rows and “*m*” columns; that is, $r_{f_{j}c_{i}}=\left[\begin{array}{ccc} r_{f_{1}c_{1}} & \cdots & r_{f_{1}c_{m}}\\ \vdots & \ddots & \vdots \\ r_{f_{l}c_{1}} & \cdots & r_{f_{l}c_{m}} \end{array}\right]$.

*Step 2*. Sort *r*_*f*_*j*_*c*_*i*__ into descending order for each class *c*_*i*_, *i* = 1 ⋯ *m*.

*Step 3*. Let the weight of feature *f*_*j*_ for class *c*_*i*_ be *w*_*f*_*j*_*c*_*i*__.

For each class *c*_*i*_, *i* = 1 ⋯ *m*.

 { Consider that “*l*” is the number of features in the dataset.
 Assign the weight “*l*” for the feature *f*_*j*_, which contains the highest value of *r*_*f*_*j*_*c*_*i*__.
 That is, *l* = max⁡{*r*_*f*_*j*_*c*_*i*__} = *w*_*f*_*j*_*c*_*i*__, for the feature *f*_*j*_.
 And assign the weight “*l* − 1” for the feature *f*_*j*_, which contains the next highest value of *r*_*f*_*j*_*c*_*i*__, and so on.
 That is, *l* − 1 = nextmax⁡{*r*_*f*_*j*_*c*_*i*__} = *w*_*f*_*j*_*c*_*i*__, for the feature *f*_*j*_.
 }. 

*Step 4*. Compute the overall weight for each feature using the equation

(2) $$\begin{array}{c} w_{f_{j}}=\frac{\sum_{i=1}^{m}w_{f_{j}c_{i}}r_{f_{j}c_{i}}}{\sum_{i=1}^{m}w_{f_{j}c_{i}}};\;j=1\cdots l. \end{array}$$

*Step 5*. Rank the features, according to the overall weight *w*_*f*_*j*__.

*Step 6*. Select top “*s* = log_2_⁡*l*” number of features based on the overall weight *w*_*f*_*j*__.

## 4. Experimental Evaluation

This section illustrates the evaluation of proposed MFSS algorithm in terms of the various evaluation metrics and the number of selected features in those applications generating multidimensional datasets.

### 4.1. Dataset

In this study, five different multidimensional benchmark datasets are used to evaluate the effectiveness of proposed MFSS [31, 32].  Table 1 summarizes the details of the dataset.

### 4.2. Evaluation Metrics

In this study, multidimensional classification with super classes (MDCSC) algorithms is used, namely, Naive Bayes, J48, IBk, and SVM. The evaluation metrics of a classification model on MDD is entirely different from the binary classification [33]. The accuracy of a classification model on a given test set is the percentage of test set that is correctly classified by the classier [34, 35]. Various evaluation metrics for multidimensional classification is available in the literature Hamming loss (HL), Hamming score (HS) precision, recall, *F*_1_, exact match (EM), and zero-one loss (ZOL) [33, 36–38].

### 4.3. Results and Discussion

This section explores the inferences of the proposed MFSS and classification algorithms which are adopted in this study. A proposed MFSS algorithm uses threshold “*s* = log_2_⁡*l*” to select the top features, where *l* is the number of features in the data set [39–41]. In our experiment, various evaluation metrics, namely, Hamming loss, Hamming score, exact match, and zero-one loss are calculated before feature selection (BFS) and after applying the proposed MFSS for each of the four classifiers, namely, J48, Naive Bayes, SVM, and IBk for MDCSC. In this work Hamming score and exact match are used to evaluate the effectiveness of the proposed MFSS [33, 37].

**Figure 3:**
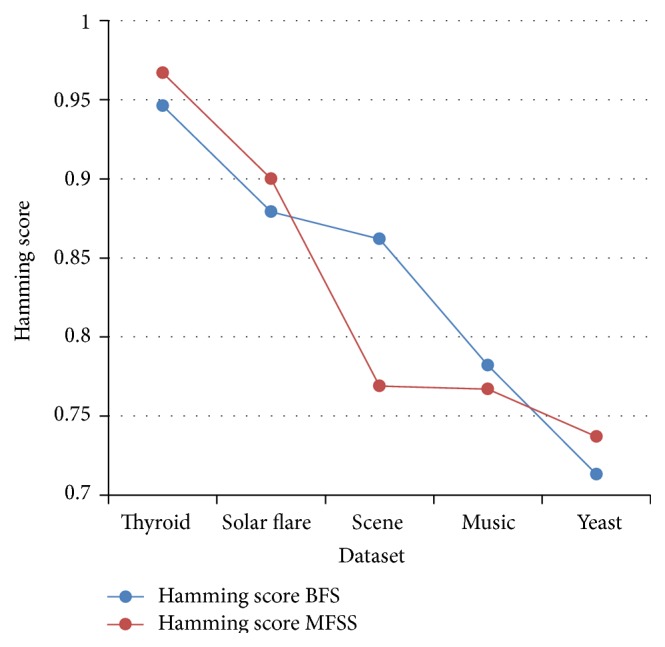
Hamming score-Naive Bayes.

**Figure 4:**
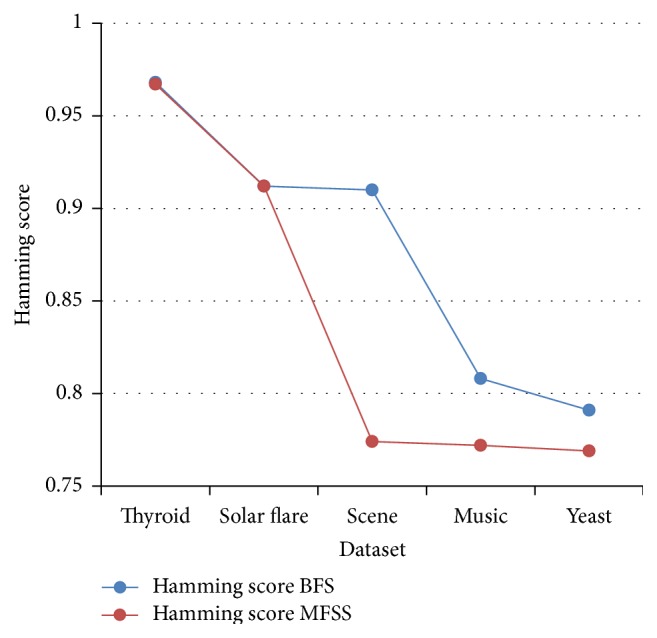
Hamming score-SVM.

**Figure 5:**
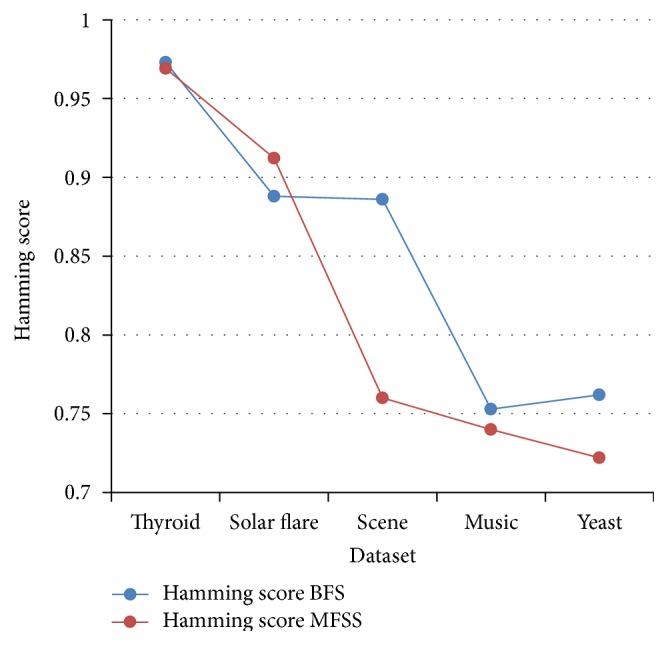
Hamming score-IBk.

**Figure 6:**
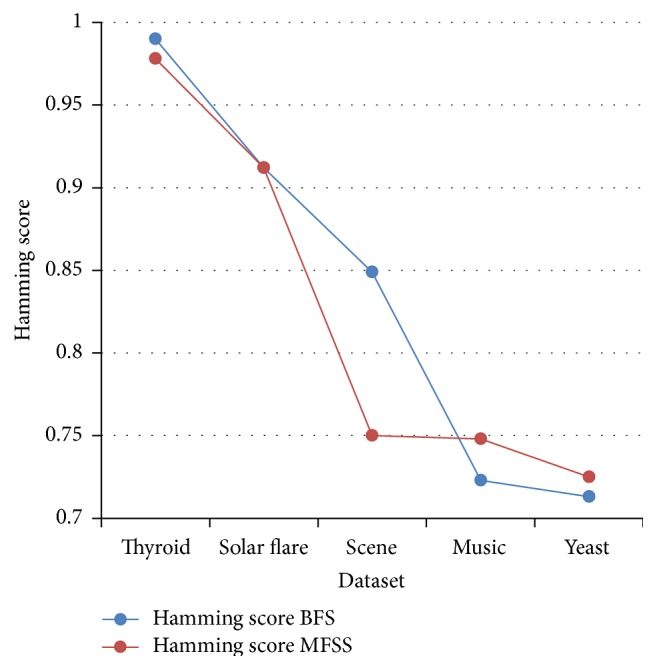
Hamming score-J48.

**Figure 7:**
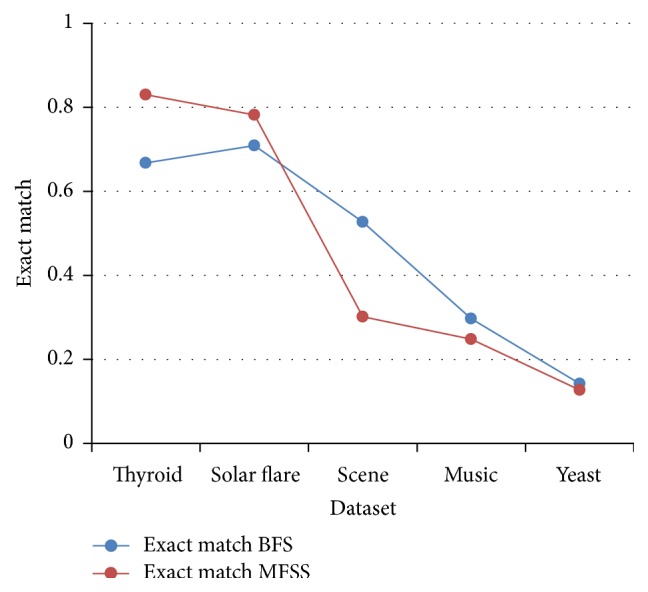
Exact match: Naive Bayes.

**Figure 8:**
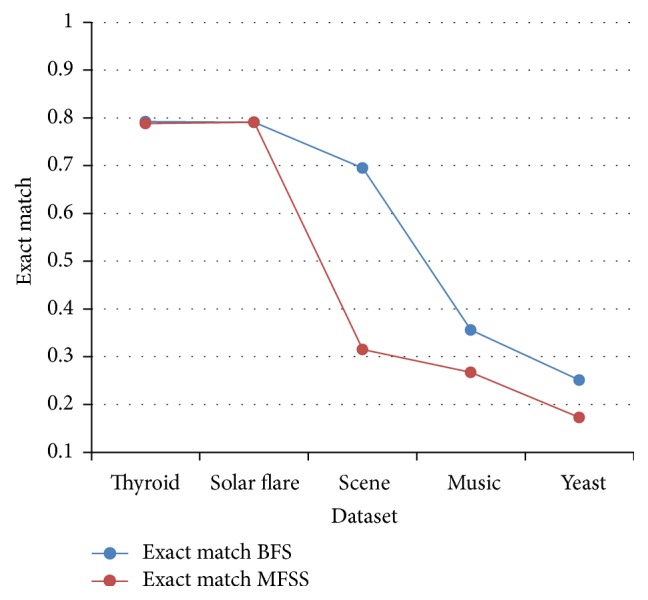
Exact match-SVM.

**Figure 9:**
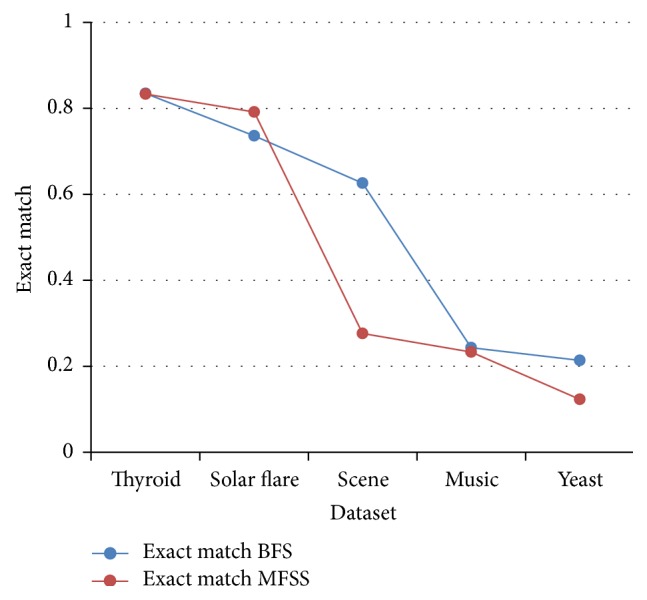
Exact match-IBk.

**Figure 10:**
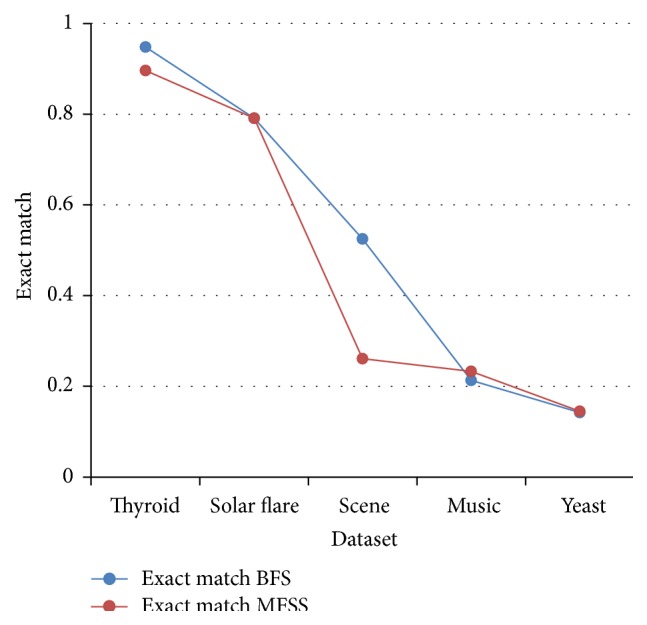
Exact match-J48.

Tables 2, 3, 4, and 5 show the experimental results of five datasets for the four classifiers J48, Naive Bayes, SVM, and IBk for raw and selected features using the proposed MFSS. Hamming loss is the fraction of misclassified instance, label pairs. It is a loss function and it is inferred that before and after applying the proposed MFSS it is nearer to zero. Figures 3, 4, 5, 6, 7, 8, 9, and 10 show the relationship between the BFS and MFSS for the evaluation metrics HS and EM for the four classifiers.

Hamming score is the accuracy measure in the multilabel setting. The highest Hamming score was 99% before feature selection (BFS) and 97.8% after applying MFSS obtained using J48 compared with the other algorithms. An exact match is the percentage of samples that labels correctly classified. The highest exact match was 94.8% before feature selection (BFS) and 89.6% after applying MFSS obtained using J48 compared with the other algorithms. For solar flare dataset highest Hamming score was 91.2% before and after applying the MFSS obtained using J48 and SVM compared with the other two algorithms. For scene dataset highest Hamming score was 91% before feature selection (BFS) and 77.4% after applying MFSS obtained using SVM. For music dataset highest Hamming score was 80.8% before feature selection (BFS) and 77.2% after applying MFSS obtained using SVM. For yeast dataset highest Hamming score was 79.1% before feature selection (BFS) and 76.9% after applying MFSS obtained using SVM.

Also, it is inferred that the exact match was nearer before BFS and after applying MFSS for four classifiers for the four datasets, namely, thyroid, solar flare, music, and yeast dataset. But for scene dataset the exact match is very less after applying the MFSS for all the four classifiers. Compared with other three algorithms SVM performs well for all the five datasets. From Figures 3, 4, 5, 6, 7, 8, 9, and 10 it is inferred that the proposed MFSS is superior to another regarding the aspects of Hamming score and exact match. Also MFSS achieves slightly poor exact match on the scene dataset for all the four classifiers.

Proposed algorithm needs to be validated by comparing the results of classifier before and after feature selection using statistical methods [42]. Correlation analysis is a technique used to measure the strength of the association between two or more variables. Correlation coefficient values always lie between −1 and +1. If the value is positive it indicates that the two variables are perfectly associated with positive linear and the value is negative, and it indicates that two variables are perfectly associated with negative linear. If the values are zero, there is no association between the variables. Evans classified the correlation coefficient into five categories such as very weak, weak, moderate, strong, and very strong [43].  Table 6 gives the details of Evans correlation coefficient classification. Pearson's correlation coefficient (*r*) is given by

(3) $$\begin{array}{c} r_{ap}=\frac{n\left(\sum ap\right)-\left(\sum a\right)\left(\sum p\right)}{\sqrt{\left[n\sum a^{2}-{\left(\sum a\right)}^{2}\right]\left[n\sum p^{2}-{\left(\sum p\right)}^{2}\right]}}, \end{array}$$

where *a* is metrics before feature selection (BFS) and *p* is metrics of proposed MFSS

The correlation coefficients between BFS and MFSS for the evaluation metrics, Hamming score, and exact match are depicted in Table 7. It indicates that the strength of association between the BFS and MFSS is very strong for all the four classifiers (*r* = 0.93, 0.868, 0.868, and 0.930 for HS and *r* = 0.947, 0.909, 0.909, and 0.947 for EM) based on Evans categorization.

The paired *t*-test is used for the comparison of two different methods of measurements that are taken from the same subject before and after some manipulation. To test the efficiency of the proposed feature selection algorithm paired *t*-test is used and the results are depicted in Table 8. The paired *t*-test statistic is given by

(4) $$\begin{array}{c} t=\frac{\sum d}{\sqrt{\left(n\left(\sum d^{2}\right)-{\left(\sum d\right)}^{2}\right)/\left(n-1\right)}}. \end{array}$$

Hypothesis for evaluation of proposed MFSS: consider the following.

 
*H*_0_: there is no significant difference between the performance of the classifier before feature selection (BFS) and after applying MFSS.
 
*H*_1_: there is a significant difference between the performance of the classifier before feature selection (BFS) and after applying MFSS.

**Figure 11:**
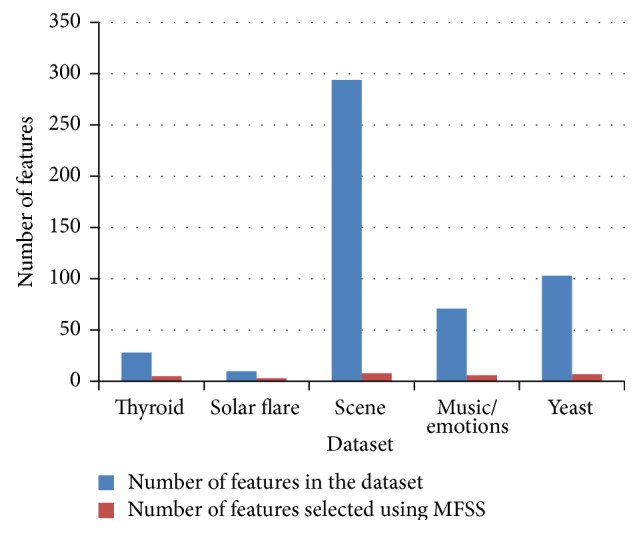
Features selected using proposed MFSS.

From the paired *t*-test for result, it is inferred that there is no significant difference between the performance of the classifier before feature selection and after MFSS for all the datasets with the critical value (2.7764, ∝ = 0.05) and (4.6041, ∝ = 0.01) for the degrees of freedom 4.  Table 9 gives the detail of features selected using the proposed MFSS.  Figure 11 shows the relationship between the features selected using BFS and MFSS. From Table 9, it can be observed that the proposed MFSS selects only a less percentage of features (minimum 3% and maximum 30%) for further analysis or to build a classifier and have the computational advantage of multidimensional classification.

Multilabel classification is categorized into two types, namely, problem transformation and algorithm adaptation. Problem transformation is to decompose the multilabel learning problem into a number of independent binary classification problems. Algorithm adaptation methods tackle multilabel learning problem by adapting popular learning techniques to deal with multilabel data directly [5]. The feature selection method is categorized into global and local. Selecting the same subset of features from all classes is called global and that identifies a unique subset of features for each class called local [44]. An existing feature selection technique in the literature concentrates only on problem transformation (i.e., first transforming the multilabel data into single-label, which is then used to select features using traditional single-label feature selection techniques) [13–16]. It does not remove all the features because the union of the identified subsets of features from all classes is equal to the full feature subset [44].

An existing feature selection technique is compared with the proposed MFSS in terms of time complexity for further analysis or to build classifier in the multilabel setting which is depicted in Table 10. “*m*” is the number of classes, “*l*” is the number of features, and “*s*” is the number of features selected using proposed MFSS in the MDD. From Table 10, the time complexity is high when the existing feature selection techniques used are compared with the proposed MFSS for further analysis or to build a classifier. Existing feature selection algorithm is suitable only for single label dataset; therefore multidimensional dataset is transformed into single label using problem transformation for feature selection. It results in “*m*” feature subset after problem transformation (i.e., a relevant feature subset for each class) but MFSS results only in a single unique feature subset. It is computationally high and complex because of “*m*” times required for further analysis or to build a classifier. Algorithm adaptation methods deal with multilabel data directly, and it requires only one feature subset for further analysis or to build a classifier. The highlight of proposed MFSS is that it yields only a single unique feature subset. Also the proposed MFSS algorithm is suitable for both problem transformation and algorithm adaptation and has great potentials in those applications generating multidimensional datasets.

To diagnose a disease, the physician has to consider many factors from the data obtained from the patients. Most researchers' aim is to identify the predictors which are used for diagnosis and prediction. The most important predictor is always increasing the predictive accuracy of the model. To diagnose the thyroid disease, physicians use the most important clinical experiments TSH, TT4, and T3. Experiment result of proposed MFSS shows that T3, FTI, TT4, T4U, and TSH are the top ranked feature. This reveals that the selected features obtained from the proposed method are same as the clinical experiments used by specialists to diagnose thyroid diseases. In almost all cases, classification results obtained using the proposed MFSS were significantly better than using the raw features. In conclusion, the study results indicate that the proposed MFSS is an effective and reliable feature subset selection method without affecting the classification accuracy even for the least number of features for the multidimensional dataset.

## 5. Conclusions

The prime aim is to select the optimum single subset of the features of the MDD for further analysis or to design a classifier. It is a challenging task to select the features with the interaction between feature and class in the MDD. In this paper, an efficient and reliable algorithm for feature subset selection from MDD based on class-feature interaction weight is proposed and the effectiveness of this algorithm is verified by statistical methods. The proposed method consists of three phases. Firstly, for each class feature-class correlation is calculated to identify the importance of feature for each class. Secondly, the weight is assigned to features based on the feature-class correlation for each class. Finally the overall feature weight is calculated based on the proposed weight method and selects the single subset “*s* = log_2_⁡*l*” number of features for further analysis or to design a classifier. The proposed MFSS algorithm selects only a less percentage of features (minimum 3% and maximum 30%) and yields unique feature subset for further analysis or to build a classifier and has the computational advantage of multidimensional classification. The experimental results of this work (MFSS) on five multidimensional benchmark datasets have improved prediction accuracy by considering only the least number of features. The proposed MFSS algorithm is suitable for both problem transformation and algorithm adaptation. Also, it reveals some interesting conclusion that the proposed MFSS algorithm has great potentials in those applications generating multidimensional datasets.

## Figures and Tables

**Table 1:** Details of the dataset used in experiments.

Dataset	Number of instance	Number of features	Number of target classes
Thyroid	9172	29	7
Solar flare	1389	10	3
Scene	2407	294	6
Music	593	72	6
Yeast	2417	103	14

**Table 2:** Evaluation metrics for J48 algorithm.

Metrics	HS		EM		HL		ZOL	
Dataset	BFS	MFSS	BFS	MFSS	BFS	MFSS	BFS	MFSS
Thyroid	**0.99**	**0.978**	0.948	0.896	0.01	0.022	0.052	0.104
Solar flare	**0.912**	**0.912**	0.791	0.791	0.088	0.088	0.209	0.209
Scene	0.849	0.75	0.525	0.261	0.151	0.25	0.475	0.739
Music	0.723	0.748	0.213	0.233	0.277	0.252	0.787	0.767
Yeast	0.713	0.725	0.142	0.145	0.287	0.275	0.858	0.855

**Table 3:** Evaluation metrics for Naive Bayes algorithm.

Metrics	HS		EM		HL		ZOL	
Dataset	BFS	MFSS	BFS	MFSS	BFS	MFSS	BFS	MFSS
Thyroid	0.946	0.967	0.668	0.83	0.054	0.033	0.332	0.17
Solar flare	0.879	0.9	0.709	0.782	0.121	0.1	0.291	0.218
Scene	0.862	0.769	0.527	0.302	0.138	0.231	0.473	0.698
Music	0.782	0.767	0.297	0.248	0.218	0.233	0.703	0.752
Yeast	0.713	0.737	0.142	0.127	0.287	0.263	0.858	0.873

**Table 4:** Evaluation metrics for SVM algorithm.

Metrics	HS		EM		HL		ZOL	
Dataset	BFS	MFSS	BFS	MFSS	BFS	MFSS	BFS	MFSS
Thyroid	0.968	0.967	0.792	0.788	0.032	0.033	0.208	0.212
Solar flare	**0.912**	**0.912**	0.791	0.791	0.088	0.088	0.209	0.209
Scene	**0.91**	**0.774**	0.695	0.315	0.09	0.226	0.305	0.685
Music	**0.808**	**0.772**	0.356	0.267	0.192	0.228	0.644	0.733
Yeast	**0.791**	**0.769**	0.251	0.173	0.209	0.231	0.749	0.827

**Table 5:** Evaluation metrics for IBk algorithm.

Metrics	HS		EM		HL		ZOL	
Dataset	BFS	MFSS	BFS	MFSS	BFS	MFSS	BFS	MFSS
Thyroid	0.973	0.969	0.834	0.833	0.027	0.031	0.166	0.167
Solar flare	0.888	0.912	0.736	0.791	0.112	0.088	0.264	0.209
Scene	0.886	0.76	0.626	0.276	0.114	0.24	0.374	0.724
Music	0.753	0.74	0.243	0.233	0.247	0.252	0.757	0.767
Yeast	0.762	0.722	0.214	0.123	0.238	0.278	0.786	0.877

**Table 6:** Evans correlation coefficient classification.

Correlation coefficient value	Strength of correlation
0.80–1.00	Very strong
0.60–0.79	Strong
0.40–0.59	Moderate
0.20–0.39	Weak
0.00–0.19	Very weak

**Table 7:** Correlation between BFS  and  MFSS for four classifiers.

Metrics	J48	Naive Bayes	SVM	IBk
Hamming score	0.914	0.867	0.801	0.859
Exact match	0.943	0.908	0.853	0.878

**Table 8:** Paired *t*-test results of different evaluation metrics before and after applying MFSS for four classifiers.

Metrics	J48	Accept/reject *H*_0_	Naive Bayes	Accept/reject *H*_0_	SVM	Accept/reject *H*_0_	IBk	Accept/reject *H*_0_
Hamming score	0.675	Accept	0.376	Accept	1.549	Accept	1.239	Accept
Exact match	1.111	Accept	0.166	Accept	1.577	Accept	1.110	Accept

**Table 9:** Features selected using proposed MFSS.

Dataset	Number of features in the dataset	Number of features selected using MFSS	Percentage of selected features using MFSS
Thyroid	28	5	18
Solar flare	10	3	30
Scene	294	8	3
Music/emotions	71	6	8
Yeast	103	7	7

**Table 10:** Comparison of time complexity.

Existing feature selection techniques in the literature	Proposed MFSS
*O*(*nc*_*i*_fs_*i*_)	*O*(*nc*_*i*_fs_*o*_)

fs_*i*_: feature subset for class  *c*_*i*_  after problem transformation, *i* = 1 ⋯ *m*; “*m*”: the number of classes.

fs_*o*_: optimal single unique feature subset using proposed MFSS for all the “*m*” classes.
